# Equine mesenchymal stem cell derived extracellular vesicle immunopathology biomarker discovery

**DOI:** 10.1002/jex2.89

**Published:** 2023-05-28

**Authors:** David E. Reynolds, Phoebe Vallapureddy, Renee‐Tyler T. Morales, Daniel Oh, Menghan Pan, Uday Chintapula, Renata L. Linardi, Angela M. Gaesser, Kyla Ortved, Jina Ko

**Affiliations:** ^1^ Department of Bioengineering University of Pennsylvania Philadelphia Pennsylvania USA; ^2^ Department of Clinical Studies New Bolton Center, School of Veterinary Medicine, University of Pennsylvania Philadelphia Pennsylvania USA; ^3^ Department of Pathology and Laboratory Medicine University of Pennsylvania Philadelphia Pennsylvania USA

**Keywords:** biomarker discovery, equine, extracellular vesicle, immunomodulation, mesenchymal stem cell, therapeutic

## Abstract

The use of mesenchymal stem cells (MSCs) in human and veterinary clinical applications has become a subject of increasing importance due to their roles in immunomodulation and regenerative processes. MSCs are especially relevant in equine medicine because they may have the ability to treat prevalent musculoskeletal disorders, among other conditions. However, recent evidence suggests that the components secreted by MSCs, particularly extracellular vesicles (EVs), are responsible for these properties. EVs contain proteins and nucleic acids, which possess an active role in intercellular communication and can be used as therapeutics. However, because the intersection of equine veterinary medicine with EVs remains a relatively new field, there is a demand to identify biomarkers that can discern and enrich for therapeutic EVs, progressing their clinical efficacy. In this study, we identified and characterized 84 miRNAs, between three equine donors involved in immunomodulation in cell and EV subjects. We discovered distinct groups of shared miRNAs, like miR‐21‐5p and miR‐451a, that are abundant and enriched between the donors’ EVs, respectively. By mapping and comparing the MSC‐EV miRNA expression, we discovered many pathways that are involved in immunomodulation and tissue regenerative processes related to equine clinical applications. Therefore, the miRNAs highlighted in this article can be used as valuable biomarkers for screening MSC‐derived EVs for potential equine therapy.

## Graphical abstract

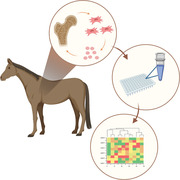

## 1 INTRODUCTION

Over the past decade, mesenchymal stem cells (MSCs) have gained popularity in the fields of medicine, bioengineering and biotechnology (Benayahu, 2022; Pittenger et al., 2019). They are found in several types of tissue including bone marrow, adipose, placenta, muscle and umbilical cord tissue (Andrzejewska et al., 2021). In the presence of inflammatory cytokines, MSCs demonstrate immunomodulating properties, particularly the ability to inhibit inflammation and promote anti‐inflammatory responses (Han et al., 2019; Li & Hua, 2017; Qi et al., 2018). The ability of MSCs to differentiate into several lineages, coupled with their roles in immunomodulation and reducing inflammation in damaged tissue, sparked their translation into clinical applications (Han et al., 2019; Qi et al., 2018; Williams & Ehrhart, 2022). For example, they have been investigated for their potential in assisting regenerative processes, including repairing damage in musculoskeletal tissue, cardiac systems, the liver, cornea and trachea (Han et al., 2019). MSCs have also been studied for their ability to improve deficits and other symptoms associated with central nervous system diseases, which include several types of neurological disorders and injury to the spinal cord (Andrzejewska et al., 2021; Han et al., 2019). The therapeutic use of MSCs in oncogenesis has also been explored, in which MSCs have been reported to suppress and incite apoptosis of cancer cells and initiate anti‐tumour immune responses (Hmadcha et al., 2020; Lee & Hong, 2017). The abundance of MSC sources and their vast utility have given rise to their candidacy in many applications, with more yet to be explored.

Considering MSCs’ strong capabilities and involvement in immunomodulation, they possess extraordinary potential in veterinary medicine, especially with equine subjects. Genetic predispositions, as well as environmental variables, place equine species at higher risk for orthopaedic diseases such as osteochondrosis and degenerative joint diseases (Metzger & Distl, 2020). Due to the chronic nature of these orthopaedic disorders and their career‐ending potential in sporting horses, strong regenerative therapies are warranted in equine veterinary medicine (Ribitsch et al., 2021; Schnabel et al., 2013). For instance, the use of a carpal osteochondral fragment model demonstrated that bone marrow (BM)‐MSCs may reduce synovial effusion and inflammation in injured joints associated with equine osteoarthritis (OA) (Schnabel et al., 2013). Others have shown that equine MSCs may be applied in the treatment of other conditions, including endotoxemia, inflammatory bowel disease, and several respiratory and reproductive disorders (Cequier et al., 2021; MacDonald & Barrett, 2020). Equine MSCs have also been investigated for the treatment of integumentary disorders and wound healing, though the effects of MSCs on wound healing were previously shown to be more significant in vitro than in vivo (Caruso et al., 2022; Cequier et al., 2021). MSCs display broad potential in equine species, but their immunomodulating properties may be affected by high passage numbers, indicating that lower passage numbers of the cells may be needed for them to reach their full therapeutic potential (Connard et al., 2021). The present challenges associated with equine health present a unique opportunity to exploit the properties of MSCs for both clinical and economical benefits.

Despite the perceived involvement of MSCs in immunomodulation, their delivery to target destinations remains low when administered. This suggests that the tropic factors secreted by MSCs, collectively labelled the secretome, are likely responsible for these effects (Gowen et al., 2020; Kearney et al., 2022; Williams & Ehrhart, 2022). The secretome contains mediators and extracellular vesicles (EVs), the latter of which include exosomes and microvesicles (Arévalo‐Turrubiarte et al., 2022; Kearney et al., 2022; Soukup et al., 2022). The use of EVs confers benefits over the use of the whole secretome as they protect their contents, proteins and nucleic acids (DNA, RNA), from degradation and do not require toxic cryopreservatives (Soukup et al., 2022). Compared to their donor cells, EVs have a higher safety profile, lower immunogenicity and can cross biological barriers (Arévalo‐Turrubiarte et al., 2022; Frisbie et al., 2009; Hmadcha et al., 2020; Hotham et al., 2021). Whilst these factors improve MSC‐EVs’ argument as a tool for therapy, their EV pool is quite large and heterogenous, making screening arduous. In equine veterinary medicine, filtering remains even more difficult, as the scope of known biomarkers is quite limited. However, several groups have confirmed that the overexpression and upregulation of miRNAs, like miR‐155, miR‐146a, miR‐200a and miR‐223 in equine individuals are indicators of infection response in equine and dramatically increase immunomodulatory effects of equine MSC‐derived EVs (Ibrahim et al., 2021; Tavasolian et al., 2020). Unfortunately, though, there still remains no clear or collective outlook on all the potential biomarkers involved in equine immunomodulation, which would improve equine MSC‐derived EV therapeutics significantly.

Herein, we sought to characterize the immunopathology landscape of equine‐derived MSC‐EVs. More specifically, we analysed 84 miRNAs from EVs and cells involved in the immunomodulation between three equine donor subjects. We discovered separate groupings of common miRNAs that are abundant and enriched in the donors’ EVs, and uncovered multiple pathways involved in immunomodulation and tissue regeneration processes in relation to horse therapeutic applications by mapping and comparing the MSC‐derived EV miRNA expression levels. To further breakdown the miRNA landscape, we also reviewed the bottom enriched EV miRNAs. We uncovered the majority of these biomarkers to have negative roles in cell proliferation and promoting tumour progression, corroborating the MSC‐EVs primary involvement in immunomodulation. To confirm the miRNA expressions reported between the cells and EVs were highly correlated, we looked for variance between the two populations. We found that cell and EV miRNA expression levels were highly positively correlated. However, between individual donor cells and EVs, we identified that the EV‐EV donor miRNA profiles were less heterogeneous than the cell‐cell expression levels. Overall, we uncovered numerous miRNAs that can be employed as indicators for evaluating MSC‐derived EVs for prospective equine treatment.

## 2 RESULTS

### 2.1 EV characterization

**FIGURE 1:**
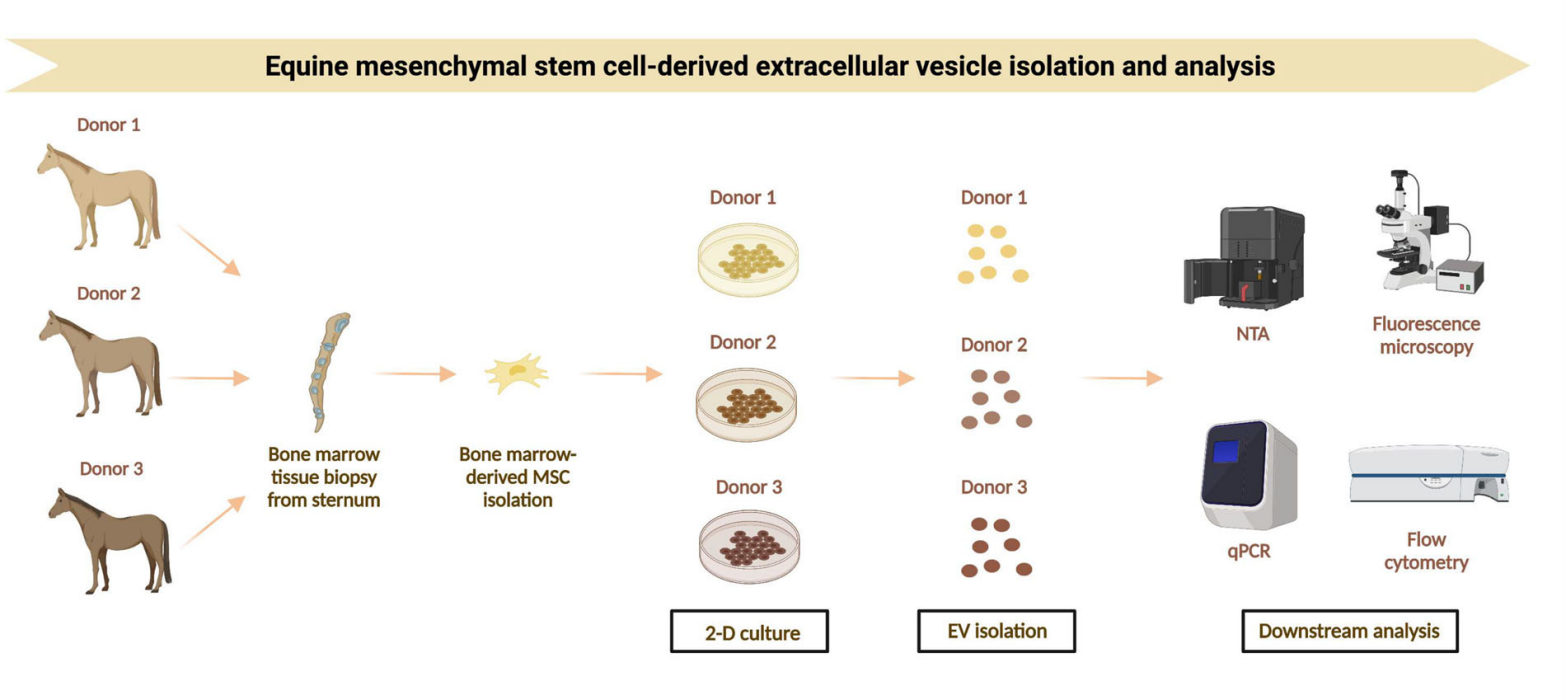
Schematic for equine BM‐MSC‐derived EV isolation and analysis. The BM‐MSCs were derived from three different equine donors. The tissue‐derived BM‐MSCs were then dissociated into single cells, cultured, and expanded. Once confluent, the EVs were isolated through ultracentrifugation, stored, and prepared for NTA and PCR.

**FIGURE 2:**
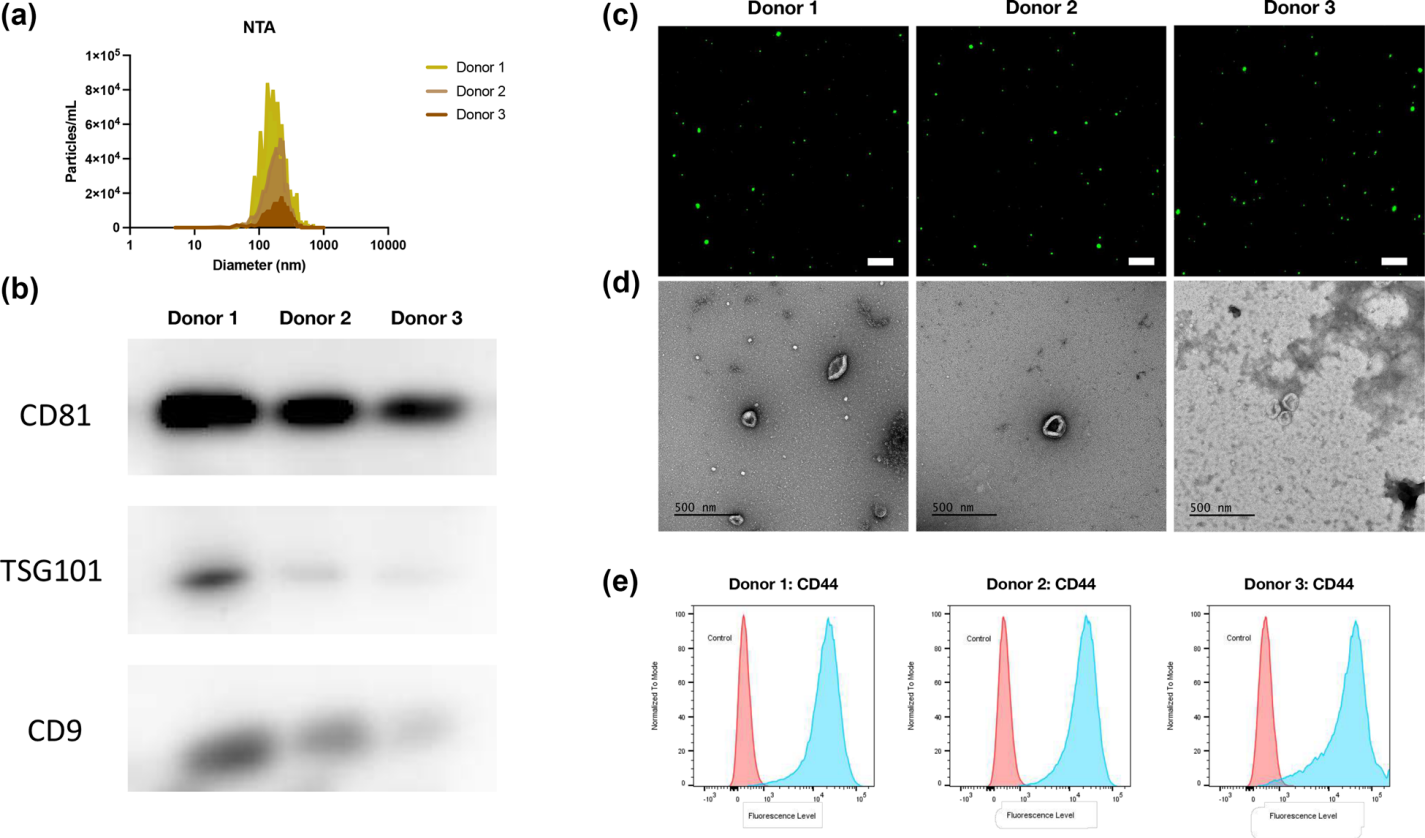
MSC‐derived EV Characterization. (a) Graph representation of NTA from isolated EVs from all three donors. (b) Western blot of CD81, TSG101, and CD9 proteins from the three donors. (c) Fluorescence imaging of Calcein‐Green Am labelled EVs on PTFE printed slide (Scale bar = 1 μm). (d) TEM images of the donors’ EVs (Scale bar = 500 nm). (e) Cell flow cytometry with CD44 primary antibody (blue) and negative control (red).

The donated bone marrow‐derived MSC (BM‐MSC) were expanded and prepared for EV isolation. For purification, the EVs were isolated through ultracentrifugation and stored in accordance with our previous work (Ko et al., 2020, 2021) (Figure 1). To verify the size distribution between the isolated EVs, the samples were measured using NTA. Based on the graphical distribution, the size spread remained homogenous between the three donors and established that the majority the EV populations are exosomes (50–150 nm) and microvesicles (100–1000 nm) (Figure 2a). For further confirmation, the EVs were stained with Calcein Green AM. Because Calcein AM remains non‐fluorescent until passing through the lipid bilayer, Gray et al. (2015) reported its effectiveness in labelling intact EVs. Thus, we chose to stain and image 10^7^–10^8^ EVs/donor on a polytetrafluoroethylene (PTFE) printed glass slide for EV isolation confirmation (Figure 2b). Comparatively, the size distribution remained indistinguishable between the three donors (<250 nm). With transmission electron microscopy (TEM), the size distribution and morphology were further assessed. Based on the images, the size of the three donors remained below 250 nm, and their morphology showed concave, oval‐shaped particles, which are expected to be EVs (Figure 2d). For EV protein quantification, CD81, TSG101 and CD9 were assessed with a western blot. To establish the existence of EVs in a biological sample, a western blot is used to determine the presence of distinctive EV‐associated proteins, like CD81, TSG101 and CD9. Based on the western blot, all three donors’ protein presence remains apparent and homogeneous (Figure 2b).

For preliminary immunomodulation verification, we investigated a cell surface adhesion reporter, CD44, among the three donor equine cells. CD44 is a cancer stemness marker that is strongly implicated in immunomodulation, as Connard et al. (2021) demonstrated by assessing the immunomodulatory capabilities of horse BM‐MSCs by continuous passaging. Through flow cytometry, we discovered that the anti‐CD44 antibody had a 50‐fold higher mean fluorescence signal compared to the negative control (no primary antibody) for our cell populations (Figure 2e). Between the individual cell groups, their fold differences remains homogenous between all three donors. This information confirms the donor equine BM‐MSC possess phenotypic immunomodulatory characteristics.

### 2.2 Comparison of immunopathology miRNA profiles

**FIGURE 3:**
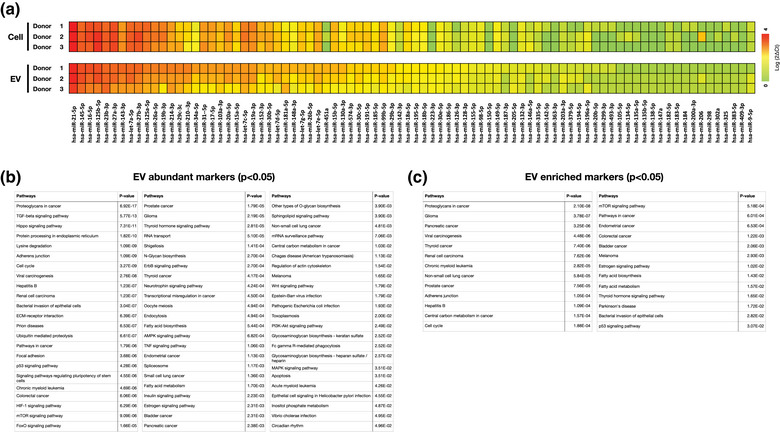
A miRNA immunopathology PCR panel was used to evaluate purified EVs and cultured BM‐MSCs from all three donors. (a) The panel includes 84 miRNA assay targets, that were normalized to Donor 1′s EVs and individually converted to a heat map based on expression levels. (b) We compared the signaling pathways between the 10 most highly expressed and shared miRNAs between the three donors. A total of 69 pathways were significantly activated (*p* < 0.05) among the equine donors. (c) We compared the signaling pathways between the 10 most highly enriched and shared miRNAs between the three donors. A total of 26 pathways were significantly activated (*p* < 0.05) among the equine donors.

Both purified EVs and cultured BM‐MSCs from all three separate donors were assessed through a miRNA immunopathology PCR panel (Qiagen). The ready‐to‐use panel is composed of 84 miRNA assay targets, as well as spike‐in control and reference miRNA assays. Although the panel is specifically designed for human immunopathology, the same individual human primer sets have been reported cross‐reactive with equine subjects by Qiagen. Thus, the human immunopathology plate can be used for overviewing the equine immunopathology landscape. By normalizing the miRNA expressions to Donor 1′s EV expression profiles, we produced a heat map consisting of a distribution from low (green) to high (red) miRNA expression, providing a whole picture of miRNA expression levels and the heterogeneity between each miRNA across all three donors (Figure 3a).

To breakdown the heat map, we compared the top 10 most highly expressed EV miRNAs (miR‐21‐5p, miR‐145‐5p, miR‐16‐5p, miR‐125b‐5p, miR‐23b‐3p, miR‐27a‐3p, miR‐143‐3p, et‐7a‐5p, miR‐27b‐3p and miR‐27b‐3p) between the three donors (Figure 3b). To ensure these top 10 reported abundant markers were within the EV membrane, we treated donor 1′s EVs with RNase A before RNA extraction. RNase A is commonly used for breaking down extracellular RNA, making it a reliable reagent for differentiating between intravesicular and extracellular RNA in EVs. Between treatment and no treatment, the relative difference between the top 10 abundant markers remained closely similar (Figure S1). This entails that there are no significant differences between the 10 reported markers and their relative abundance with and without RNAse treatment. Thus, the majority of the previously reported markers are inside the EVs. We identified that 69 signalling pathways were significantly activated (*p* < 0.05) with Diana Tools mirPath software (Vlachos et al., 2015). The pathways remain diverse, but many of them, such as the TGF‐beta signalling pathway, lysine degradation, ECM‐receptor interactions, adherent junctions and focal adhesions, are involved in the control of normal and OA joints (Kang et al., 2016; Mok & Urschel, 2020; Shen et al., 2014; Van Der Kraan, 2018). Other pathways and their roles, including mTOR and HIF‐1 signalling pathways, have been investigated in exercised and sepsis‐related laminitis equine subjects, respectively (Hauss et al., 2021; Pawlak et al., 2014). Renal cell carcinoma, thyroid cancer, endometrial cancer and other cancer pathways have also been reported in equines (Chaffin et al., 1990; Dalefield & Palmer, 1994; Wise et al., 2009). Together, the reported pathways corroborates the 10 reported abundant EV miRNA to be significant in equine immunopathology.

Because we were also interested at the dispersion of packaged miRNAs in the donors’ EVs, pathways were analysed with the 10 most enriched miRNA in EV compared to those in cells (miR‐451a, miR‐223‐3p, miR‐205‐5p, miR‐150‐5p, miR‐126‐3p, miR‐142‐3p, miR‐142‐5p, miR‐363‐3p, miR‐210‐3p and miR‐34a‐5p) (Figure 3c). We identified 26 activated signalling pathways (*p* < 0.05). Comparatively, proteoglycans in cancer, renal cell carcinoma and thyroid cancer were all significantly triggered between both the abundant and enriched groups. The enriched EV group, though, appeared to have far less activated pathways. For instance, there were almost no related pathways involved in regenerative processes, like the regulation of OA joints. Not to mention, the glioma pathway had a larger presence in the enriched group, but is not frequently reported among equines. Because there remains limited information behind the selective packaging into EVs, it remains difficult to understand the reasoning why the groups’ pathways are different. For instance, EVs might not selectively sort/package specific cargo from their mother cells. Thus, supporting the importance and efforts of understanding the mechanisms behind EVs packaging and involvement in immunopathology.

To validate if these signalling pathways are induced by the miRNAs in the MSC‐EVs, we exposed the HEK293T cells to donor 1′s EVs and characterized their mRNA profiles with Human Adheren Junction (Qiagen) and Cancer PathwayFinder (Qiagen) panels. Because the majority of these pathways are related to cancer and tissue regeneration, we chose mRNA panels that would provide comprehensive information about their activation. Based on the cancer panel, we uncovered that there was up‐regulation in several markers, including GADD45 and CASP9, whilst there was down‐regulation in markers like APAF‐1 and MCM2 (Figure S2). Based on the literature, GADD45 and CASP9 are responsible for DNA repair and preventing apoptotic death early in development, respectively (E Tamura et al., 2012; Li et al., 2017) On the other hand, APAF‐1 and MCM2 promote tumour progression (Shakeri et al., 2017; Sun et al., 2022). For the adheren junction panel, DSC1 and DSG3 were up‐regulated, whilst ZYX and PKP3 were down‐regulated (Figure S3). Reports indicate that DSC1 and DSG3 are critical for intracellular adhesions and membrane trafficking, whilst ZYX and PKP3 have potential involvement in carcinogenesis in many cell types (Harrison et al., 2016; Moftah et al., 2017; Partynska et al., 2022; Ruan et al., 2021). Based on these findings, we can confirm that the MSC‐EVs are therapeutic in nature, as the up and down‐regulated mRNAs from the treated cells were therapeutic and carcinogenic, respectively.

### 2.3 miRNA biomarker analysis

**FIGURE 4:**
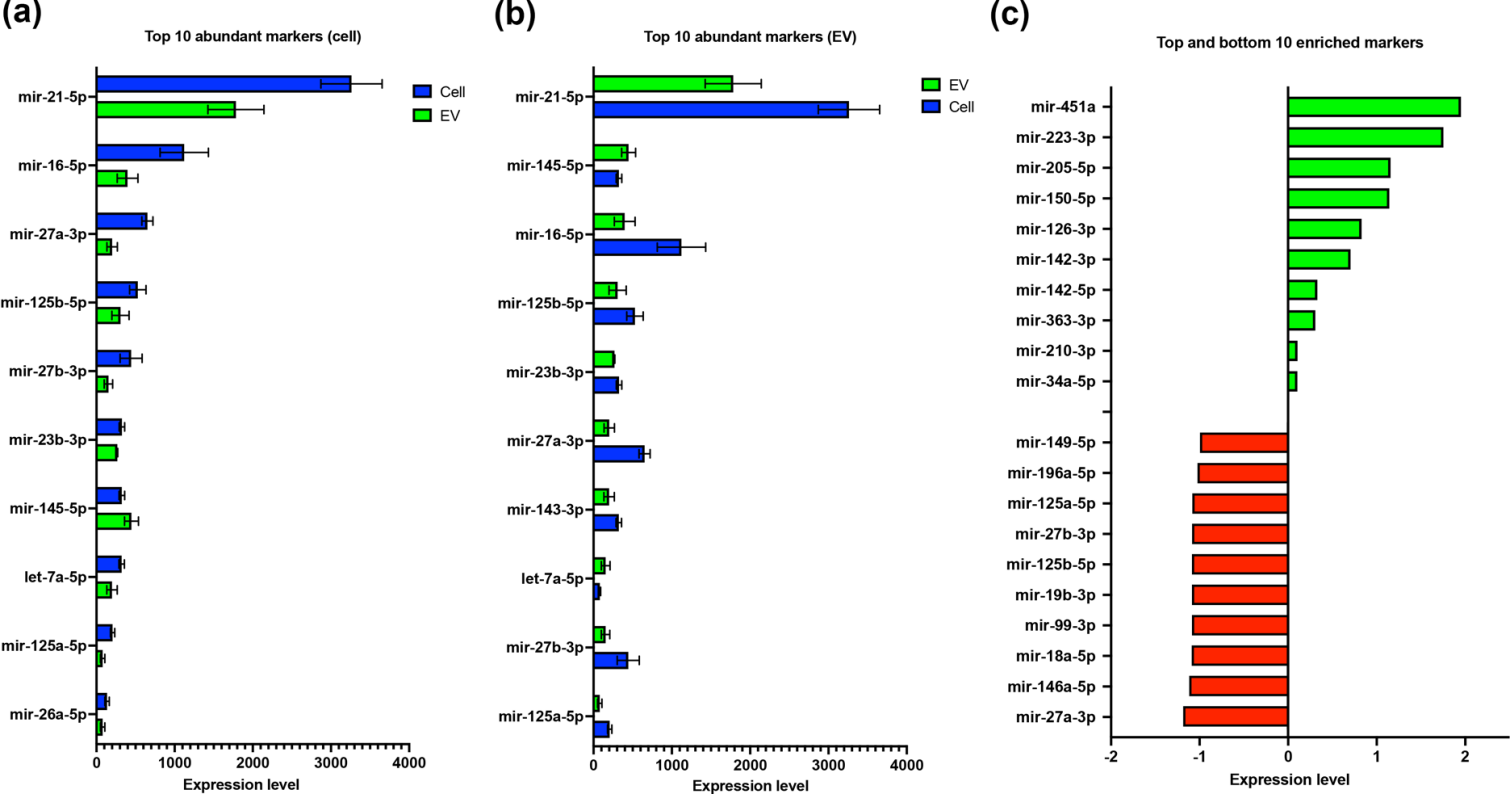
RNA profile analysis of donors’ cells and EVs. (a) Grouped bar graph of the 10 most abundant miRNAs with respect to cell normalization. (b) Grouped bar graph of the top 10 abundant miRNAs with respect to EV normalization. (c) Bar graph representation of the top and bottom 10 most enriched miRNAs in the EVs.

To break down the miRNA profiles between the cells and EVs, we plotted the 10 most abundant markers for both groups (Figure 4a,b). Interestingly, miR‐21‐5p had the highest expression between both cell and EV populations. However, there remains very limited literature on its therapeutic significance in equines. Most findings, among human subjects, reveal miR‐21‐5p importance as a biomarker for numerous types of cancers and hypersensitivity, and cellular processes like apoptosis and proliferation (Lao et al., 2021; Liu et al., 2021; Yan et al., 2019; Zhang et al., 2021). Other markers, such as miR‐27a‐3p, miR‐27b‐3p, miR‐143‐3p, also have minimal preexisting information on its immunomodulatory significance with equines and their EVs. However, miRNAs, such as miR‐145‐5p and miR‐23b‐3p have been reported as prospective biomarkers for monitoring acute pain in equines (Lecchi et al., 2018). Not to mention, miRNA‐16 and miRNA‐125b were reported to be down‐regulated in OA synovial fluid samples and highly expressed in sarcoid tissue, respectively (Baker et al., 2022; Unger et al., 2019). Whilst a handful of these miRNAs have been shown to be immunomodulatory, the newly reported ones, such as miR‐21‐5p, miR‐27a‐3p, miR‐27b‐3p and miR‐143‐3p, serve as novel screening candidates for equine treatment. Although these miRNAs are critical for MSC regulation in equine, they have not been reported among other equine cell types. However, they have been reported in other human cells, like fibroblast, epithelial cells, and immune cells (Kang et al., 2021; Naito et al., 2014; Potenza et al., 2017; Song et al., 2020).

Outside the abundant markers, the top and bottom 10 enriched markers were also assessed (Figure 4c). The majority of the top enriched markers, including miR‐205‐5p, miR‐150‐5p and miR‐126a‐3p, have not been reported as potential biomarkers for immunopathology in equines. However, miR‐451a and miRNA‐223‐3p have been cited to be highly upregulated in metabolic unbalanced and respondent in infected equines, respectively (Cappelli et al., 2021; Lange‐Consiglio et al., 2018). On the other hand, bottom enriched markers, such as miR‐27a‐3p, miR‐18a‐5p, miR‐99‐3p and miR‐125b, have been recorded for their negative roles in cell proliferation and promoting tumour progression (Chen et al., 2021; Liang et al., 2017). Because the majority of the bottom enriched markers are involved in producing a cancerous phenotype, it can be assumed that the MSC‐EVs are therapeutic in nature.

### 2.4 Heterogeneity between EVs and cells

**FIGURE 5:**
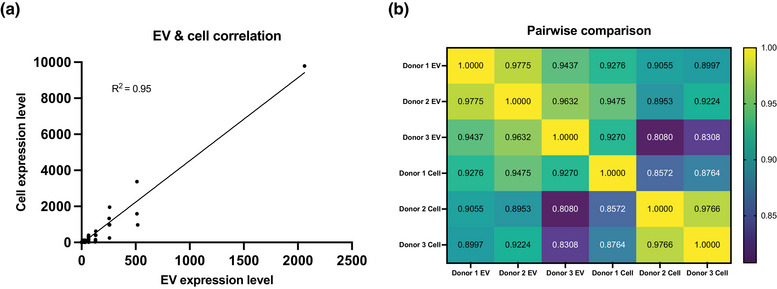
Heterogeneity between donors’ cells and EVs. (a) Cell and EVs RNA expression values plotted against each other for correlation measurement. (b) Pairwise heat map of donor‐to‐donor RNA expression value correlation measurement. Purple and yellow represent low and high correlation, respectively.

We examined the cell and EV populations to establish that the miRNA expressions observed were substantially associated. The miRNA expression values for both cells and EVs were averaged between all three donors and then plotted against one another. We found that cell and EV miRNA expression levels were highly positively correlated (*R* ^2^ = 0.95) (Figure 5a). We generated a pairwise heat map versus the individual donor cells and EVs to further break down the heterogeneity (Figure 5b). We discovered that the EV‐EV profiles are less heterogeneous than the cell‐cell expression levels. For instance, the donors’ EV‐EV and cell‐cell miRNA profiles range from 0.94 to 1 and 0.86 to 1, respectively. Interestingly, the EV‐mother cell profiles were similarly heterogeneous to the cell‐cell miRNA profiles, 0.81–0.95. Overall, the heterogeneity between the cells and EVs remained low, supporting the conservation of their miRNA profiles.

## 3 DISCUSSION

The ability of MSCs to differentiate into different lineages and influence immunomodulation processes has motivated their broad exploration in clinical applications. MSCs have become increasingly relevant in veterinary medicine involving equine subjects, given that they are at increased risk for musculoskeletal disorders. MSC‐derived EVs are now widely believed to be responsible for the properties of their donor cells, and previous studies have demonstrated that bone marrow‐derived extracellular vesicles may reduce inflammation in equine OA. Unfortunately, though, there is still no clear or collective view on all of the possible biomarkers implicated in horse immunomodulation, which would considerably enhance equine MSC‐derived EV therapies. Herein, we discovered several miRNAs that may not be only applicable for the treatment of OA, but the alleviation of several different disorders in equine subjects, like sepsis and cancer. We also discovered miRNAs, such as miR‐21‐5p, miR‐27a‐3p, miR‐27b‐3p and miR‐143‐3p, that have not been reported as immunomodulatory markers for screening for potential therapeutic MSC‐EVs. Besides the identification of new biomarkers, we also assessed the miRNA profiles pathways and the heterogeneity between the donors cells and EVs. Overall, we provided a comprehensive panel and overview of potential biomarkers for equine subjects, advancing therapeutics in equine veterinary medicine.

Although this work was targeted at equine veterinary medicine, the data and workflow are translatable for human subjects. For instance, the reported miRNAs in this paper are originally targeted for humans and have been reported as biomarkers for several human disorders and diseases; thus, reconfirming their importance in human diagnostics and therapeutics. Whilst there are many clear advantages to this work, there remains a few limitations. For instance, whilst qPCR can be convenient, this method remains limited to the assessment of only a few genes. To provide a more comprehensive overview and increased dynamic range of the MSC‐EV equine immunopathology, RNA‐seq would be more suitable. Thus, in the future, we plan to use RNA‐seq for further biomarker discovery and validation in our equine MSC‐EVs. Additionally, to resolve the heterogeneity of MSC and MSC‐EV, more donors will be included in future studies. In the future, we aim to test these particular MSCs on equines with OA and other disease phenotypes, providing validation of their immunomodulation capabilities.

## 4 EXPERIMENTAL SECTION/METHODS

### 4.1 Cell isolation

This methodology is in accordance with the recommendations of the Institutional Animal Care and Use Committee (IACUC) at the University of Pennsylvania: Bone marrow was collected aseptically from the sternebrae of systemically healthy horses (7–12 years old) using an 11‐gauge Jamshidi bone marrow biopsy needle (VWR Scientific, Bridgeport, NJ) and 60 mL syringe containing 10,000 U of heparin, 30 mL of bone marrow was aspirated (Figure S4). Bone marrow samples were processed via density centrifugation with Ficoll‐Paque Plus (GE Healthcare, Chicago, IL, USA) prior to seeding into flasks containing medium consisting of Dulbecco's Modified Eagle Medium (DMEM) with 1 g/L of D‐glucose, 2 mM L‐glutamine and 1 mM sodium pyruvate (ThermoFisher Scientific, Hampton, NH), penicillin (100 U/mL)‐streptomycin (100 μg/mL) solution (Invitrogen, Carlsbad, CA), 10% foetal bovine serum (FBS) (VWR Life Science Seradigm, VWR, Radnor, PA), and basic fibroblastic growth factor (bFGF, 1 ng/mL) (Invitrogen, Carlsbad, CA). Medium was changed every 48 h. BM‐MSC were cultured in 37°C/5% CO2 and passaged when they reached ∼80% confluency, using Trypsin‐EDTA Cell Dissociation Reagent (ThermoFisher Scientific, Waltham, MA). Cell number and viability of BM‐MSC‐passage (P) 2 were determined using the Cellometer Auto 2000 Cell Viability Counter (Nexcelom Bioscience, Lawrence, MA) and ViaStain AOPI staining solution (Nexcelom Bioscience LLC, Lawrence, MA).

### 4.2 Cell culture & EV isolation

BM‐MSCs were cultured in a 150 mm cell culture dish and expanded to 6–8 dishes for EV isolation. The cells were cultured and passaged in DMEM (10% FBS, 1% penicillin). Once the cells were confluent, the media was replaced to exosome‐depleted DMEM (5% exosome‐depleted FBS, 1% penicillin). After 48 h from media exchange, the collected supernatant was spun at 400 *g* for 5 min and filtered with a 0.22 μm vacuum filter to remove cellular debris. The supernatant was centrifuged (Beckman Coulter) at 100,000 *g* for 70 min at 4°C twice. The EV pellet was resuspended in PBS and aliquoted and stored in −80°C.

### 4.3 EV characterization (Qubit, NTA)

Two different techniques were used to characterize the EVs. Qubit (Thermo Fisher) was used to assess the protein content, and nanoparticle tracking analysis (NTA) was used to determine how many particles were present. Thermo Fisher's protein assay kit was used for Qubit, and measurement was done in accordance with the manufacturer's instructions. The measurement for NTA was carried out at University of Pennsylvania School of Veterinary Medicine Extracellular Vesicle Core (ZetaView by ParticleMetrix). The analysis employed the identical parameters (sensitivity of 75 and shutter of 75).

### 4.4 EV calcein labelling

EV labelling is performed with Calcein Green AM (Thermo Fisher). EV and Calcein Green AM (1 mM) aliquots are thawed at room temperature. The Calcein Green AM stock aliquot is diluted to 10 μM in PBS with 1.0E7–1.0E8 EVs. The working solution is then incubated at 37°C for 20 min. After incubation, the working solution is pipetted on to individuals wells of a PTFE printed slide as 20 μL aliquots. Then, the EVs are allowed to settle on the glass at RT for 45 min. The wells are then washed three times with PBS and immediately imaged.

### 4.5 Cell flow cytometry

Cells were collected fresh before staining. The cells were stained with 5 μg/mL of the primary antibody, CD44 (BioXCell), at RT for 30 min. After two washes with PBS, the cells were labelled with 2 μg/mL of the secondary antibody (Thermo) at RT for 20 min. Before flow cytometry, the cells were washed twice. The flow data was analysed using FlowJo.

### 4.6 Western blot analysis

Extracellular vesicle samples (10^8^–10^9^ particles) containing 2× Laemmli loading buffer [Bio‐Rad Cat.#161‐0737] were loaded into a 4%–12% gel pre‐casted gel (NuPAGE, Invitrogen, total volume of 20–30 mL per well) in a non‐reducing condition. Precision Plus Protein Kaleidoscope Prestained Protein Standards (Bio‐rad, #1610375, 5 mL/well) and PageRuler Prestained Protein Ladder (Thermo Scientific, #26616, 5 mL/well) were used as molecular weight markers. The electrophoresis was initially run at 80 V for 20 min and then at 150 V for 60 min. The proteins were transferred from the gel to a polyvinylidene difluoride (PVDF) membrane (Immobilon‐P, Millipore, #IPVH00010) at 70 V for 2 h. After transfer, the membrane was briefly rinsed in tris‐buffered saline solution and blocked with 5% dry skim milk for 2 h at room temperature before incubation with a primary antibody overnight at 4°C. Primary antibodies included anti‐CD9 (Biolegend, #312102), anti‐CD81 (Santa Cruz Biotechnology, sc‐166029 HRP), and anti‐TSG101 (Millipore Sigma, #AV38773). Then the membrane was washed with TBS+Tween 20 (TTBS) at room temperature (three times for 5 min each) and then incubated with horseradish peroxidase (HRP) secondary antibody for 1 h at room temperature. Secondary antibodies included goat anti‐mouse for CD9 and CD81 (Biolegend, #405306) and donkey anti‐rabbit for TSG101 (Biolegend, #406401). The membrane was washed with TTBS for 2 h, changing the wash every 15–20 min, and then incubated with Immobilon Forte Western HRP Substrate (Millipore, #WBLUF0100) for two min at room temperature for chemiluminescence detection. Detection was performed with Cytiva Amersham ImageQuant 800 with 30–120 s of exposure time.

### 4.7 Transmission electron microscopy (TEM)

TEM images were taken at the Electron Microscopy Resource Laboratory, University of Pennsylvania. A 3 μL volume of sample was applied to a thin carbon grid that was glow discharged for 2 min using a Pelco Easyglow instrument. Following it, a 3 μL of freshly made 2% Uranyl acetate stain solution was then applied to the sample holding grid and incubated with the sample for 2 min. Excess samples and stains were blotted away with a Whatman filter paper leaving a thin layer of stained particles on the grid. The staining process was repeated one more time with 3 μL of the stain solution. The stain was then blotted away, and the grid was left to dry until imaging. TEM micrographs were collected using a Tecnai T12 TEM microscope at 100 KeV. The images were recorded on Gatan Oneview 4Kx4K camera. Each image was collected by exposing the sample for 4 s, and a total of 100 dose‐fractionated images were collected and placed into a single micrograph. The data was collected at −1.5 to 2 microns under focus at 30K – 40 K magnification.

### 4.8 Quantitative polymerase chain reaction (qPCR)

RNA was purified with the miRNeasy Tissue/Cells Micro Kit (Qiagen; 217084). RT‐PCR was performed using both cell and EV RNA. The miRCURY LNA RT Kit (Qiagen; 339340) was used for RT‐PCR. Using the kit, the EV RNA was mixed with reagents and run in a SimpliAmp Thermal Cycler (Applied Biosystems) as per the manufacturer's protocol. The qPCR master mix is from the miRCURY LNA SYBR Green PCR Kit (Qiagen; 339345), which consists of 2X miRCURY SYBR Green Master Mix and a ROX Reference Dye. The 96‐well Human Immunopathology Focus V2, miRCURY LNA miRNA Focus PCR Panel (Qiagen; 339325 YAHS‐204YA) was used for qPCR. Each well is prefilled and labelled with the primers. 10 μL of the qPCR master mix was added to each well. For every qPCR experiment, 40 cycles were run using a 7500 Real‐Time PCR machine (Applied Biosystems).

### 4.9 RNase A treatment

EVs from donor 1 were treated with RNase A (ThermoFisher; EN0531) at a concentration of 0.2 μg/mL at 37°C for 30 min. RNase inhibitor (Takara; 2313A) was then added at a concentration of 1 U/μL was added to the solution at 37°C for 10 min to stop the reaction. RNA was subsequently purified with the miRNeasy Tissue/Cells Micro Kit (Qiagen; 217084). RT‐PCR was performed using EV RNA. The miRCURY LNA RT Kit (Qiagen; 339340) was used for RT‐PCR. Using the kit, the EV RNA was mixed with reagents and run in a SimpliAmp Thermal Cycler (Applied Biosystems) as per the manufacturer's protocol. The qPCR master mix is from the miRCURY LNA SYBR Green PCR Kit (Qiagen; 339345), which consists of 2X miRCURY SYBR Green Master Mix and a ROX Reference Dye. The 96‐well Human Immunopathology Focus V2, miRCURY LNA miRNA Focus PCR Panel (Qiagen; 339325 YAHS‐204YA) was used for qPCR. Each well is prefilled and labelled with the primers. 10 μL of the qPCR master mix was added to each well. For every qPCR experiment, 40 cycles were run using a 7500 Real‐Time PCR machine (Applied Biosystems).

### 4.10 Cell pathway activation experiment

HEK293T cells were cultured in a 96‐well plate in DMEM (10% FBS, 1% penicillin). Once 80% confluent, the media was replaced with exosome‐depleted DMEM (5% exosome‐depleted FBS, 1% penicillin). The cells were then treated with varying dosages (0, 0.5, 1, 2, 4 μg) of donor 1′s EVs. After 12 h of incubation with the EVs, RNA was extracted from the cells with Trizol Reagent (ThermoFisher; 15596018), labelled with GlycoBlue Coprecipitant (ThermoFisher; AM9515), and treated with 2U of TURBO DNase (ThermoFisher; AM2238) at 37°C for 30 min. EDTA was then added to inactivate the TURBO DNase, with a concentration of 15 mM for 10 min at 75°C. The iScript cDNA Synthesis Kit (BioRAD; 1708890) was used for RT‐PCR. With this kit, the cell RNA was mixed with its reagents and run in a SimpliAmp Thermal Cycler (Applied Biosystems) as per the manufacturer's protocol. The qPCR master mix includes SsoAdvanced Universal SYBR Green Supermix Kit (BioRAD; 1725270) and nuclease‐free water. The 96‐well Human Adherens Junction (Qiagen; 330231 PAHS‐146ZA) and Cancer PathwayFinder (Qiagen; 330231 PAHS‐033ZA) RT^2^ Profiler™ PCR Arrays were used for qPCR. Each well is prefilled and labelled with the primers. 10 μL of the qPCR master mix was added to each well. For every qPCR experiment, 40 cycles were run using a 7500 Real‐Time PCR machine (Applied Biosystems)

## CONFLICT OF INTEREST STATEMENT

The authors declare no competing financial interest.

## Supporting information

Supporting Information
