# Supplementary material for: Equine mesenchymal stem cell derived extracellular vesicle immunopathology biomarker discovery
Source: J Extracell Biol. 2023 May 28;2(6):e89. doi: 10.1002/jex2.89 (PMC11080797; doi:10.1002/jex2.89)
Supplement: Supplementary file 1 — Supporting Information [file JEX2-2-e89-s001.pdf]

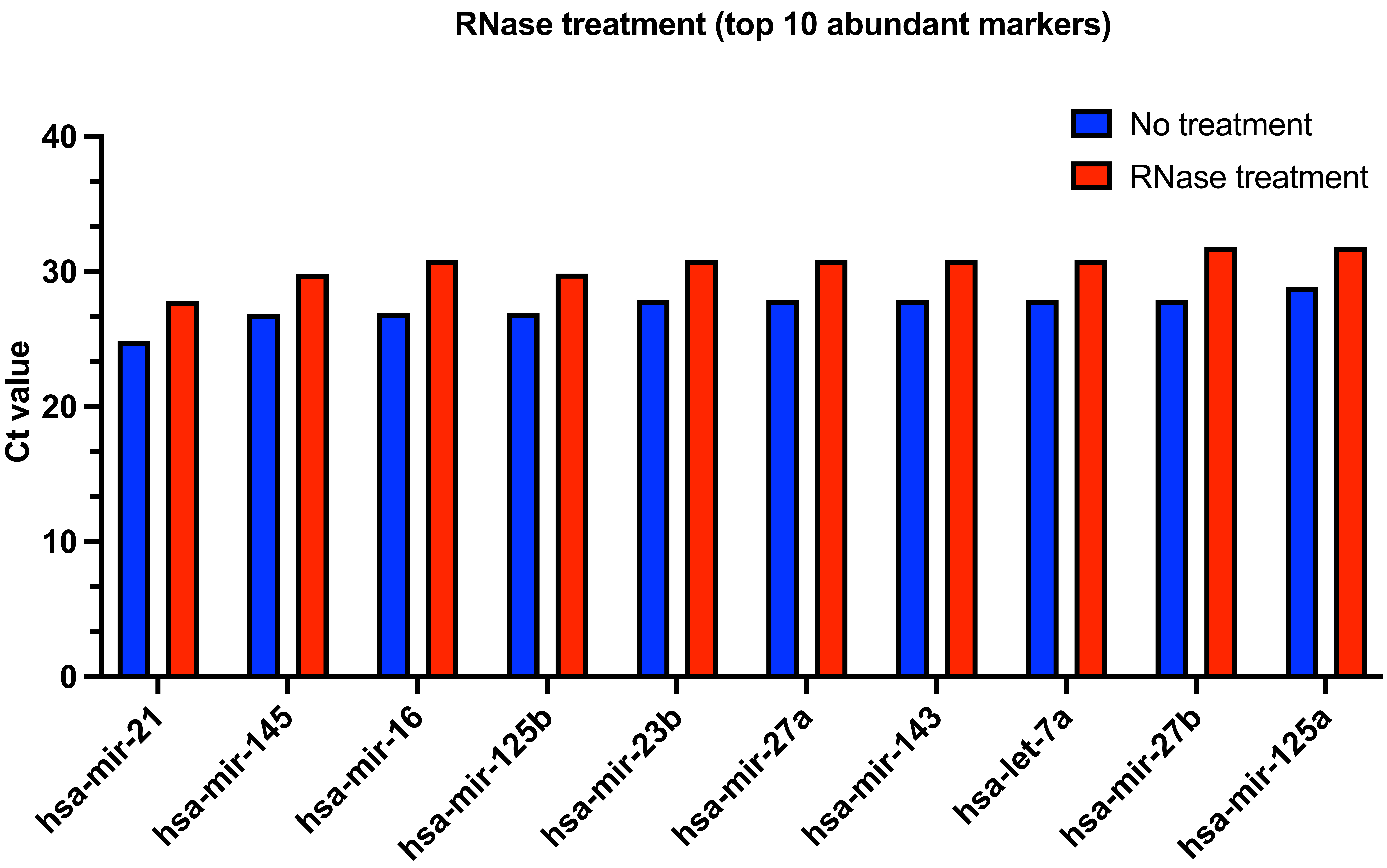

Supplementary Figure 1. Top 10 abundant makers with (red) and without (blue) RNase A treatment.

Cancer PathwayFinder Fold Change

Target	0.5 ug	1 ug	2 ug	4 ug
ACLY	0.24843	0.49680	0.49301	0.49782
ACSL4	0.14026	0.49842	0.49613	0.49419
ACTB	0.12463	0.24818	0.49562	0.49693
ADM	0.24903	1.99267	0.99175	0.99239
ANGPT1	0.12527	0.99527	0.12328	0.00372
ANGPT2	0.25057	8.01263	0.99438	0.01489
APAF1	0.12400	2.00469	1.99327	0.00186
ARNT	0.13256	0.49769	0.49598	0.99289
ATP5A1	0.12497	0.49914	0.99652	0.99251
AURKA	0.12399	1.00524	0.49999	1.00296
B2M	0.12402	0.49708	0.49698	0.99501
BCL2L11	0.24208	0.49868	0.49577	0.49721
BIRC3	0.49568	1.04727	0.96172	2.18025
BMI1	0.48993	0.50013	0.24898	0.50037
CA9	0.06204	1.06731	2.28416	4.59795
CASP2	0.24753	0.49581	0.49310	0.49708
CASP7	0.50258	0.49952	0.24839	0.49858
CASP9	0.01488	1.00549	1.00099	2.00816
CCL2	0.24973	0.41565	0.05956	2.00522
CCND2	0.12447	1.00447	0.99442	0.99829
CCND3	0.12409	1.00203	0.49543	0.49710
CDC20	0.24775	1.00167	0.99579	1.99808
CDH2	0.06287	0.49979	0.49743	0.49505
CFLAR	0.24821	0.50038	0.49933	0.99850
COX5A	0.24479	0.49339	0.49496	1.99652
CPT2	0.12464	0.50272	0.50112	0.50261
DDB2	0.12397	0.49570	0.99517	0.99430
DDIT3	0.06168	2.00180	3.99963	8.02410
DKC1	0.49576	0.99720	0.99484	0.99244
DSP	0.49728	1.00011	0.99503	0.99904
E2F4	0.06217	0.49753	0.49436	0.24837
EPO	0.24886	0.49981	0.99407	0.49698
ERCC3	0.24808	0.50019	0.24792	0.49857
ERCC5	0.12408	0.99631	0.99782	0.99112
ETS2	0.24790	1.00059	0.49919	0.50046
FASLG	0.99861	1.97986	0.89472	0.97597
FGF2	0.50004	0.99931	0.99375	1.99306
FLT1	1.00184	0.50036	0.49935	0.99943
FOXC2	0.99421	0.99361	0.24703	0.99713
G6PD	0.24855	0.49847	0.49788	0.49762
GADD45G	0.24843	1.04129	1.10981	8.90231
GAPDH	0.49988	0.24832	0.49902	0.24848
GPD2	0.49570	0.49430	0.99436	0.49755
GSC	0.24772	0.49910	0.49695	0.50042
HMOX1	0.24781	0.49711	0.49461	0.49749

Target	0.5 ug	1 ug	2 ug	4 ug
HPRT1	0.12319	0.49891	0.99202	1.00039
IGFBP3	0.12559	0.49903	0.06265	0.12539
IGFBP5	0.12430	0.49798	0.49726	0.49678
IGFBP7	0.12955	0.99823	0.99891	1.99869
KDR	0.24950	1.00254	0.11259	1.00350
KRT14	0.12485	0.99609	0.12337	0.49874
LDHA	0.22003	0.50268	0.50089	0.49749
LIG4	0.24896	0.99561	0.99406	0.99742
LPL	0.06217	1.00246	0.49688	0.99103
MAP2K1	0.06217	0.49742	0.49498	1.00185
MAP2K3	0.49373	0.99998	0.99337	0.99873
MAPK14	0.49490	0.49920	0.49873	0.49864
MCM2	0.12407	0.25023	0.24696	0.24770
MKI67	0.49883	0.49836	0.49673	0.99742
NOL3	0.12455	0.99768	0.98948	0.98850
OCLN	0.00186	1.00042	1.00303	2.00777
PFKL	0.49269	0.49503	0.99383	0.49477
PGF	0.12456	0.49817	0.24909	0.24749
PINX1	0.12536	0.50093	0.50104	0.50220
POLB	0.12453	0.49878	0.49727	0.99754
PPP1R15A	0.12488	0.99854	0.99411	1.99857
RPLP0	0.24955	0.99725	0.99226	1.98638
SERPINB2	0.12460	1.98114	0.22917	0.49847
SERPINF1	0.12442	0.99600	0.98717	1.99712
SKP2	0.49849	0.49794	0.49533	0.49507
SLC2A1	0.24852	1.00105	0.99796	0.50074
SNAI1	0.24891	1.00122	0.12495	0.49985
SNAI2	0.24893	1.00220	0.99906	2.00728
SNAI3	0.24576	1.00312	0.06201	0.24968
SOD1	0.06194	1.00302	0.99704	2.00205
SOX10	0.05952	0.50093	0.02976	1.99529
STMN1	0.50090	0.25134	0.25171	0.50269
TBX2	0.50107	0.49842	0.49651	0.49676
TEK	0.50032	1.11346	1.03702	1.03571
TEP1	0.24857	0.99961	1.99641	1.99966
TERF1	0.24774	0.24847	0.24656	0.99548
TERF2IP	0.25071	1.00233	0.99625	2.00369
TINF2	0.03105	0.49954	0.49781	0.49943
TNKS	0.06239	1.00356	1.00090	0.99030
TNKS2	0.06204	0.49994	0.49899	0.99515
UQCRFS1	0.06254	0.50273	0.50134	2.01170
VEGFC	0.13687	0.99684	0.99996	1.99817
WEE1	0.24920	1.00297	0.50104	1.00523
XIAP	0.12406	0.49520	0.24751	0.49417

Supplementary Figure 2. Table comparing fold change of treatment conditions to no treatment for Cancer PathwayFinder panel.

Adheren Junction Fold Change

Target	0.5 ug	1 ug	2 ug	4 ug
ACTB	0.06253	0.50030	0.63322	0.39933
ACTN1	0.12535	0.49899	0.62671	0.39723
ACTN2	0.25020	0.99740	1.26488	0.00074
ACTN3	2.15503	1.08547	1.24821	0.86310
ACTN4	0.25022	0.49794	0.63357	0.39803
AJAP1	0.25066	1.00012	0.00490	0.19952
ANAPC1	0.12511	0.49389	0.31174	0.39739
ARF6	0.25278	1.00664	1.27798	0.80042
ARVCF	0.50017	0.50188	0.63330	0.80101
B2M	0.12559	0.50199	0.63697	0.79802
BAIAP2	0.25041	3.99955	0.03949	0.19894
CBLL1	0.12581	1.00075	1.26970	1.60118
CDC27	0.12509	0.49778	0.63115	0.78870
CDC42	0.25122	1.00248	1.26879	0.78643
CDH1	0.25288	0.50130	0.63572	0.80421
CDH2	0.25028	0.49850	0.63099	0.39006
CDH3	0.25195	0.99758	0.07945	0.40105
CDH4	1.00746	0.49930	1.26905	0.79481
CDH5	0.05824	0.24916	0.00952	0.04972
CDSN	0.12465	0.49658	0.03513	0.09938
CSNK2A1	0.50471	2.01360	2.55541	3.20452
CSNK2A2	0.25130	2.02003	2.54329	3.21591
CSNK2B	0.25063	1.99742	2.54288	3.20163
CTNNA1	0.25062	0.99950	1.26387	1.59346
CTNNA2	0.88360	1.00541	0.15249	0.79085
CTNNA3	0.13706	1.06953	0.17380	0.10938
CTNNB1	0.06264	0.49842	0.31712	0.19881
CTNND1	0.49988	0.99757	0.62693	0.79788
DLG5	1.00315	0.99957	1.26303	0.79805
DLL1	0.12474	0.49942	0.63235	0.79826
DNM1	0.50014	0.49577	0.62976	0.39541
DNM2	0.49751	0.99381	0.61923	0.19689
DOCK4	0.03006	3.99948	1.25758	0.79667
DSC1	1.00161	16.74511	1.27004	13.34713
DSC2	0.49920	0.99738	1.26066	0.79368
DSC3	0.25011	1.99975	0.63049	0.79105
DSG1	0.05956	1.99205	0.53719	0.04753
DSG2	0.12548	1.00168	0.63203	0.79561
DSG3	0.13200	1.02142	1.20917	3.54121
DSG4	1.12774	0.15179	0.19313	4.07889
DSP	0.50232	1.00068	1.26545	0.79545
EXOC2	0.12571	0.49980	0.63653	0.79746
FARP2	0.25102	0.50047	0.63484	0.19945
FLNA	0.24944	0.49627	0.62235	0.39878
FLNB	0.24950	2.01439	1.27053	0.79845

Target	0.5 ug	1 ug	2 ug	4 ug
GAPDH	0.06264	0.24966	0.31601	0.09906
HGS	0.12467	1.00264	0.63249	0.80020
HPRT1	0.25023	0.49896	1.26452	0.79667
IQGAP1	0.24939	2.00465	1.26430	1.60604
JUP	0.25009	2.02336	1.27461	0.80186
LMO7	0.06260	0.50103	0.31575	1.59931
MAPRE1	0.25035	1.00467	1.26808	0.79202
MAPRE2	0.24997	1.00056	0.63153	0.79833
MLLT4	0.49974	0.49653	1.26372	0.79561
NME1	0.24837	0.49585	1.26556	0.79447
NOTCH1	0.06230	0.49722	0.63009	0.39626
NOTCH2	0.25032	0.49534	1.26309	1.59596
NOTCH3	0.25019	0.99798	1.25338	0.01191
NOTCH4	0.03007	2.01218	1.26079	0.79986
P2RX6	0.02998	4.04175	0.63217	0.40194
PARD3	0.24838	0.99933	1.26353	0.79276
PERP	0.24944	2.00350	1.26705	1.57714
PIK3CG	1.00161	0.99822	1.27004	0.79934
PKP1	0.91822	0.98639	0.15131	0.46857
PKP2	0.12487	0.99921	0.63115	0.79862
PKP3	0.06270	0.98970	0.03569	0.00297
PKP4	0.25058	0.49852	0.63212	0.39426
PNN	0.12576	0.49804	0.62831	1.60313
PPAP2B	0.25092	1.00010	1.26347	0.79380
PPL	0.06218	1.00731	0.15960	0.20085
PVRL1	0.12390	1.00196	1.25841	1.59470
PVRL2	0.12459	0.99883	0.62889	0.79963
PVRL3	0.12487	0.99921	1.26740	1.59166
PVRL4	0.06016	0.48176	0.07628	0.28694
RAC1	0.03113	0.49885	0.63338	1.58989
RALA	0.25048	0.99928	1.26634	0.00019
RHOA	0.50153	0.99830	1.26178	1.59979
RPLP0	0.50363	1.00300	1.26663	1.60311
SORBS1	0.06286	1.00472	0.63693	0.80211
SSX2IP	0.12494	0.49862	0.63148	0.79500
TJP1	0.06302	0.49754	0.63585	0.80301
TLN1	0.12593	0.50230	0.63594	0.19793
TLN2	0.12453	0.99540	0.31243	0.39591
VCL	0.25028	2.02115	2.55063	1.60131
VEZT	0.06224	0.99790	0.63322	1.59549
WAS	0.12471	0.49949	0.63362	0.39763
WASF1	0.06237	0.49995	0.63457	1.59246
WASL	0.25169	1.00605	1.27537	0.79365
ZYX	0.12516	0.49924	0.63224	0.00019

Supplementary Figure 3. Table comparing fold change of treatment conditions to no treatment for Adheren Junction panel.

	Age	Gender	Breed
**Donor 1**	12	Male	Standardbred
**Donor 2**	19	Male	Thoroughbred
**Donor 3**	8	Male	Quarter Horse

Supplementary Figure 4. Table outlining the age, gender, and breed of the horses.
